# Towards an official gene nomenclature for *Populus trichocarpa*

**DOI:** 10.1093/treephys/tpaf054

**Published:** 2025-05-09

**Authors:** Susan Tweedie, Stanton Martin, Elspeth Bruford

**Affiliations:** HUGO Gene Nomenclature Committee, Department of Haematology, University of Cambridge School of Clinical Medicine, Long Road, Cambridge, Cambridgeshire, CB2 0PT, UK; Center for Bioenergy Innovation, Oak Ridge National Laboratory, 1 Bethel Valley Road, Oak Ridge, TN 37830, USA; HUGO Gene Nomenclature Committee, Department of Haematology, University of Cambridge School of Clinical Medicine, Long Road, Cambridge, Cambridgeshire, CB2 0PT, UK

**Keywords:** bioinformatics, database, gene names, gene naming, orthologs, Populus trichocarpa, paralogs, Plant Gene Nomenclature Committee

## Abstract

The HUGO Gene Nomenclature Committee (www.genenames.org), which has been naming human genes for over 40 years, has been tasked with establishing an official gene nomenclature system for the black cottonwood tree *Populus trichocarpa* (Torr. & Gray). Here, we review the factors that must be considered when establishing gene nomenclature guidelines. What makes a good gene symbol, and what lessons can be learned from other nomenclature projects? Are there particular challenges associated with naming genes in poplar species? We look at the published gene symbols for *Populus* and highlight some issues, e.g., the same symbols being used for different genes, and diverse approaches to naming in gene families. What approaches can we take to resolving such conflicts? Since community adoption is key to the success of any nomenclature initiative, we have surveyed poplar researchers for feedback on draft guidelines and discussed some of the issues raised. Finally, we discuss the sustainability of such infrastructure projects—if we build it, will they come and who will fund the ongoing work?

## Why name genes?

Gene naming is not a glamorous topic, but there is no doubt that standardized gene symbols and names play a vital role in enabling effective science communication. Short, ideally pronounceable, meaningful and hence memorable gene symbols allow us to talk about genes in a way that is simply not possible with anonymous and decidedly forgettable gene IDs. However, problems can arise when different symbols are being used for the same gene, or when the same symbols are being used for different genes. Confusing plant genes could result in years of wasted research time and money from using the wrong reagents or studying the wrong gene, as has been seen when human genes have been confused (Braschi et al. 2021). Ideally every species needs a unique set of gene symbols that are accepted and used by the research community. To maximize the chances of achieving this, the community needs a set of agreed-upon gene naming guidelines and an authority to approve and maintain the naming. For most species, this role has traditionally been taken on by model organism databases, or in the case of humans, by the HUGO Gene Nomenclature Committee (HGNC, www.genenames.org), but sadly, not every species is cared for in this way. Plants, and especially tree species, have been more neglected in this regard than most.

The relative ease and low cost of DNA (deoxyribonucleic acid) sequencing have resulted not only in more species being sequenced but more varieties and more individuals within a species. Comparative genomics is challenging without standardized naming of data (Cannon et al. 2025); in particular the ability to readily identify equivalent genes in different samples, populations and genomes can be invaluable. This prompted the team at Oak Ridge’s Center for Bioenergy Innovation to seek help assigning gene symbols to aid comparative analysis in *Populus trichocarpa*. They approached the HGNC with a challenge—could we help name genes in poplar? The HGNC is the sole authority for naming human genes and has been doing so since the 1970s, so is well placed to offer advice. This invited review discusses our approach and progress.

## How do genes get their names?

The HGNC is the only authority responsible for assigning standardized human gene nomenclature. The official HGNC website, www.genenames.org, lists the approved gene symbol and name for every human gene named to date, along with alternative names and symbols that have been published, and links to many external genomic resources. Changes to genes symbols are discouraged but when they are necessary, the original symbol (and name) are recorded as previous symbols (and names). It is HGNC-approved symbols and names that are displayed on all major resources about human genes including NCBI Gene, UCSC, Ensembl, etc. Scientists are encouraged to contact the HGNC prior to naming (or renaming) a gene and we work together to agree on appropriate nomenclature. We encourage journals both to include our guidance on gene nomenclature (Bruford et al. 2020) in their instructions to authors and not to publish new symbols without HGNC approval. For many model organisms, the role of nomenclature authority has been taken on by the model organism databases. A prime example of this is WormBase (Sternberg et al. 2024) for *C. elegans* and related nematodes. However, not all model species have such a dedicated resource and only a few exist for plants. Examples are The Arabidopsis Information Resource (TAIR) (Reiser et al. 2024) and MaizeGDB (Woodhouse et al. 2025). Different nomenclature authorities take different approaches. Some strictly favour the first published symbol, others—including HGNC—take other factors into account and place more emphasis on finding the most informative and standardized nomenclature.

HGNC has already expanded the nomenclature efforts from human to seven key vertebrate species that did not have their own dedicated nomenclature groups. This sister project, the Vertebrate Gene Nomenclature Committee (VGNC), aims to name genes in all seven core species (chimp, macaque, cattle, pig, horse, dog and cat) in line with their human orthologs and paralogs; we also accommodate gene family sets for additional vertebrates that have been submitted by experts (Jones et al. 2023). Building on this experience, our naming activities have now extended to plant species, specifically in *P. trichocarpa*, under the title of the Plant Gene Nomenclature Committee (PGNC).

Gene nomenclature comprises a gene name that describes a key feature (or features) of the gene product, paired with a gene symbol that is typically a short form or acronym of the gene name. Every gene also receives an essential and unique gene identifier. These identifiers are critical for the purposes of data mining and should be quoted in publications when a gene is first mentioned. However, for subsequent references to that gene in the context of a single publication, the gene symbol will suffice.

## Gene naming in plants

*Arabidopsis thaliana* is one of the most highly researched and published plant model organisms, and inevitably, many gene symbols used in poplar have been based on the symbols used for the Arabidopsis homologs. Arabidopsis has established naming guidelines based on agreed community standards (Meinke and Koornneff 1997), which are cited within the nomenclature portal on the TAIR website (https://www.arabidopsis.org/), and while it can be helpful to follow Arabidopsis nomenclature for poplar orthologs, there are some drawbacks. The TAIR guidelines say ‘at this time… we are not adopting a standardized system of gene nomenclature. We will concentrate our efforts on making associations of gene names and aliases so that information relating to each gene can be obtained regardless of the variable nomenclature.’ TAIR encourages authors to register gene class symbols to minimize accidental duplications in gene nomenclature; however, there are some duplications in the list of already registered symbols and in some cases both symbols have already been used for poplar homologs. Further, orthology calls between Arabidopsis and poplar are not always straightforward and some phenotype-based names from Arabidopsis may not be relevant to poplar.

Guidelines also exist for species such as wheat (Boden et al. 2023), maize (https://www.maizegdb.org/nomenclature) and rice (McCouch et al. 2008), but these are more distantly related to poplar. In some cases, such as Rosacea species (Jung et al. 2015) and grapevine (Grimplet et al. 2014), guidelines have been published, but do not yet seem to have translated into many genes actually being named—likely due to a lack of funding for such efforts. To the best of our knowledge, there is no specific guidance for naming genes in any forest tree species.

## Naming genes in *P. trichocarpa*

*Populus trichocarpa* is an important model tree species that is already associated with a substantial body of literature. It has the advantage of a mature genome reference sequence that is already on assembly version 4.1 (https://phytozome-next.jgi.doe.gov/info/Ptrichocarpa_v4_1) (Tuskan et al. 2006). The genomic data for version 4.1 and some previous assemblies can be found in Phytozome (Sreedasyam et al. 2023), the Plant Comparative Genomics portal of the Department of Energy’s Joint Genome Institute; each gene annotation is numbered relative to its position along a given chromosome with a unique ‘Potri ID’ such as Potri.014G089400. Potri IDs are used as the primary ID or included as synonyms in other databases, including Ensembl, NCBI Gene and UniProt. We will retain Potri IDs as the primary ID for *P. trichocarpa* genes in PGNC. However, ID formats have not been consistent across all previous assemblies and it can be a challenge to identify genes in the current assembly with old IDs. We are also aware that some published genes are no longer represented in the current genome assembly (Wullschleger et al. 2013). Another factor that must be considered in terms of gene naming in poplar is the recent whole-genome duplication event, which resulted in duplicate copies of many genes (Tuskan et al. 2006).

As a starting point for naming in *P. trichocarpa* we reviewed the gene symbols that are already in use in databases including NCBI Gene (Brown et al. 2015), Ensembl (Dyer et al. 2025), UniProt (UniProt Consortium 2025) and Phytozome. With the exception of tRNA genes and genes encoded by the chloroplast genome, very few standardized *P. trichocarpa* gene symbols are displayed in any of these databases. To augment this initial set, we harvested over 3000 symbols used in *P. trichocarpa* publications. The resulting list highlighted a number of key issues: multiple symbols for the same gene, e.g., *WND1A* (Zhong et al. 2010) *VNS12* (Ohtani et al. 2011) and *SND1-A1* (Li et al. 2012) for Potri.011G153300; the same root symbol used for different gene families, e.g., TPS for trehalose-6-phosphate synthase (Yang et al. 2012) and for terpene synthase (Irmisch et al. 2014); alternative numbering schemes within gene families, e.g., NRAMPs named based on both orthology/paralogy (Pottier et al. 2022) and chromosomal location (Ma et al. 2023); various approaches to naming families and close paralogs; clashes with symbols in other species, e.g., *SOS* used for ‘salt overly sensitive’ (Tang et al. 2010) in poplar versus *SOS* referring to Ras/Rac guanine nucleotide exchange factors related to the Drosophila ‘son of sevenless’ gene (Webb et al. 1993); different symbol styles, e.g., *ASH2-1* (Zhang et al. 2024), *AMT1;1* (Wu et al. 2015), *REM1.1* (Raffaele et al. 2007), *MYB83* (Bewg et al. 2022), *MYB001* (Wilkins et al. 2009) and *LecLRK1* (Labbé et al. 2019).

Our hope is that the introduction of clear nomenclature guidelines can help improve the consistency of symbols and that, going forward, some of these issues will be less prevalent. Existing problems will need to be addressed by consultation. The use of multiple symbols for the same gene can be contentious. Researchers like to put their own ‘mark’ on a gene they have worked on, even when they already have a name! The simplest solution is to use precedence of publication, but this can result in an uninformative symbol being retained at the expense of a more descriptive symbol. In general, these cases need to be tackled on a case-by-case basis, with weight being given to systematic and informative nomenclature. HGNC, in common with other curated genomic resources, records alternative or ‘alias’ symbols so that genes can readily be identified. We acknowledge that some researchers can remain wedded to specific symbols, so we encourage authors to cite the approved symbol at least once in their papers so that they can be correctly indexed.

Using root symbols for more than one gene family or group, such as TPS for trehalose-6-phosphate synthase (Yang et al. 2012) and for terpene synthase (Irmisch et al. 2014), presents a major problem as it is a key principle that approved symbols must be unique—‘*TPS1’* cannot be approved for both trehalose-6-phosphate synthase 1 and terpene synthase 1. Who wins or should both change? In a case like this, we would consider usage of the symbols in publications, what symbols are used for orthologs in other species, are any alternative symbols in use that could be acceptable to the community.

One of the valuable features of approved gene symbols is that they are stable—if the gene model is the same or very similar, then the symbol should not change. One recurring issue relates to genome analysis papers where gene families are identified and named but not studied in detail. These papers often number gene family members in order along the chromosomes and ignore previously published nomenclature. This makes it difficult to compare genes between different species and makes no effort to group subfamilies of related genes. Worse still, there is a tendency for other authors to repeat the analysis and rename the genes based on new assemblies, resulting in the reuse of symbols for different genes. In an extreme example, Zhao et al. (2018) catalogued the genes encoding basic helix-loop-helix proteins in *P. trichocarpa* genome assembly v3.0 and named them in chromosomal order from ‘bHLH1’ (Potri.001G062900) to ‘bHLH199’ (Potri.019G099500). Ye et al. (2021) revisited this family in the v4.1 assembly in 2021 and found additional genes, but rather than retaining the same symbols for the genes present in both assemblies, they renamed the whole set by chromosome order. This resulted in every bHLH gene being assigned a different symbol, with the added confusion that these symbols had already been used for a different but related gene.

While we are in a position to offer nomenclature advice and general guidelines, with limited resources, we are unable to resolve very complex or contentious naming issues. Self-organized coordination among researchers working in the same field is undoubtedly the best approach to finding nomenclature that is acceptable to all parties. This approach was taken to resolve the multiple naming conventions for the cellulose synthase family in poplar (Kumar et al. 2009) and is strongly encouraged.

## A survey of the poplar research community

While we have thoughts on how to tackle some of the naming issues that we have identified, we felt it was crucial to get input from the poplar research community. To this end, we presented our guidelines for consideration within a short survey, which asked for opinions on a number of alternative approaches to naming. The survey was initially shared with researchers at Oak Ridge and attendees at the IUFRO Tree Biotech 2024 (https://treebiotech.org/) conference and then sent to corresponding authors that we identified as having published several articles on poplar—in total the survey was sent to over 100 researchers.

The consensus across other plant guidelines is that gene symbols should be in uppercase letters, and in human and most vertebrate species, gene symbols are uppercase. However, we noted that *P. trichocarpa* symbols are often published using a mix of cases so we asked if we should allow mixed case symbols (e.g., *UBCc* (Payyavula et al. 2009), *WOX1a* (Liu et al. 2014) and *CASTORa* (Cope et al. 2019). Close to 60% of respondents were in favor of this, so our recommendation is that they can be approved, but usually only in cases where they have already been used extensively in the literature.

We will aim for approved gene symbols to contain at least four characters and recommend that every gene symbol include a number (or letter) after the root/class symbol (e.g., *CEN1* (Sheng et al. 2023), *PHYB1* (Howe et al. 1998, Sheng et al. 2023)). However, we are aware that some shorter symbols (e.g., *LFY* (Rottmann et al. 2000)) are in common use. The survey revealed that the majority of respondents (80%) would prefer that exceptions be made to retain shorter symbols that are embedded in the literature, as sometimes happens in human gene naming.

To allow for effective searching, gene symbols should not be the same as commonly used abbreviations or words and should not be the same as symbols for unrelated genes in other species. As we have already mentioned, some root symbols used for plant genes have a different meaning in other species. So, we asked researchers about their attitude to existing symbol clashes between species. There was no consensus on this topic—half thought it was not a problem as the fields are distinct and half thought the clashing symbols should either not be approved at all or a change to the least published should at least be considered. So we will have to consider these on a case-by-case basis.

When naming large human gene families, we often recruit experts to provide input and ongoing advice (Gray et al. 2016). These ‘specialist advisors’ provide valuable insights into a research area and their endorsement of an approved nomenclature can help it become the accepted standard. Their contribution is credited on the HGNC website and they are listed as a member of our specialist advisors’ panel. As part of the survey, we asked for volunteers with expertise in specific poplar gene families and were encouraged that over half of those who responded offered their help.

We also asked, should the names of uncharacterized gene family members be qualified, e.g., include ‘(putative)’ or ‘homolog’ or ‘family member’ in the name? And if there is experimental evidence of function in an ortholog in another plant species, e.g., sphagnum, would we then drop the ‘putative’ term for the poplar gene? While some felt such an indication was unnecessary, the vast majority were supportive of adding some qualifier to the gene name when the function of the gene product has not yet been established.

## Nomenclature guidelines

We are currently formalizing our proposed guidelines for naming genes in *P. trichocarpa* and hope to share these with Populus researchers soon. The guidelines are based on nomenclature guidance published for other species and take into account some of the issues we encountered in our literature review, together with the responses we received in the survey. Further discourse with researchers prior to publication is important to ensure the guidelines align with the community’s consensus on gene naming criteria and approval. If you are interested in being part of this discussion, please contact us.

The basic guidance can be summarized as:

(i) Symbols should be composed of Roman letters and Arabic numbers
(ii) No Greek letters, Roman numerals, spaces, ‘G’ for gene
(iii) Punctuation should be limited to periods or hyphens used as separators
(iv) No reference to species, including *P. trichocarpa*, in symbols and names
(v) Where possible, related genes should be named using a common root/class symbol
(vi) Numbering should reflect homology, not chromosomal order/order of publication
(vii) Aim to approve the same symbols for orthologs across plant species

What can be done to ensure that approved nomenclature is universally accepted?

One of the most important things is awareness—everyone working in the field should know that there is now an official gene nomenclature and where to find it. It is vital that the approved gene symbols are consistently displayed by all major databases, so we will encourage as many resources as possible to incorporate the approved gene names and symbols, including Ensembl, NCBI Gene and UniProt, and any specialized database that includes *P. trichocarpa* data, e.g., Phytozome, TreeGenes (Falk et al. 2018) and Gramene (Tello-Ruiz et al. 2022).

Journals also play a key role in encouraging the use of approved nomenclature, but this is most effective when they can point authors and reviewers to a definitive source of approved nomenclature and clear naming guidelines. Ideally, no new or revised nomenclature should be published without the blessing of the nomenclature authority. We will fill this gap for *P. trichocarpa* (and potentially other species) and request that journals update their instructions to authors. At present, these instructions can be somewhat vague; e.g., *Tree Physiology* ‘follows international community guidelines’ that are ‘often based upon those developed for the model plant *Arabidopsis thaliana*’.

## Progress and future plans

We have already started populating a PGNC database with nomenclature, which can be found at the website https://plant.genenames.org. Our primary effort is focussed on naming protein-coding genes in the current reference assembly (*P. trichocarpa* v4.1); each gene is identified in the PGNC website using the current Potri ID.

**Figure 1:**
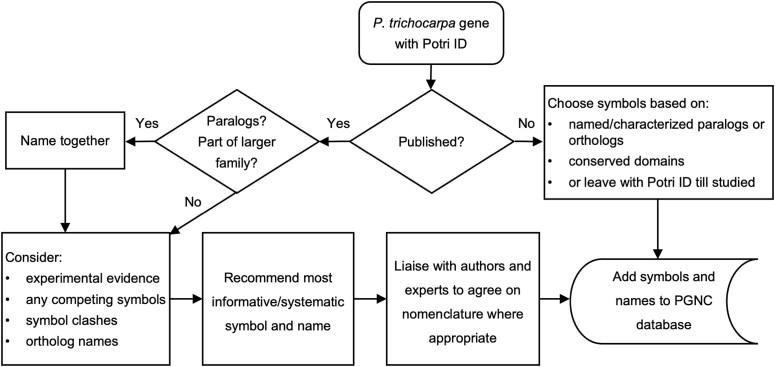
A summary of the key steps in our workflow for assigning gene nomenclature in *P. trichocarpa*.

A summary of the key steps in our workflow for assigning gene nomenclature is presented in Figure 1. As we aim to name related genes with a shared root/class symbol, a key consideration is whether a gene has paralogs. Where possible, published nomenclature is adopted, but we also weigh this against the need to avoid problematic symbols and the desire to have consistent names for orthologs where appropriate.

So far, we have focussed on approving names for well-published poplar genes. We have also worked with our specialist advisor, David Nelson, to name the set of Cytochrome P450 genes (CYPs) (Tuskan et al. 2006). The CYP symbols are used universally across all species (Nelson 2018) and David had already liaised with researchers to name some CYP genes in *P. trichocarpa* (Irmisch et al. 2013). The genes encoding ribosomal proteins are another example of a large set that will require expert input (Lan et al. 2022). We will also contact all those who offered assistance with other gene groups via the survey.

We have also started to name genes that have clear one-to-one orthology across a wide range of species, including Arabidopsis, human and yeast—typically these genes encode proteins involved in fundamental processes such as cell division. The survey revealed support for taking a phylogenetic approach to naming, and it makes sense for these genes to be named in line with every other species. However, given the lack of standardization, caution is required when basing poplar nomenclature solely on Arabidopsis and it is vital to check the consistency of Arabidopsis gene nomenclature. For example, TAIR’s primary Arabidopsis symbols for the functionally redundant ACETOACETYL-COA THIOLASE paralogs *AACT1* (AT5G47720) and *ACAT2* (AT5G48230) do not share a root symbol, and confusingly, *AACT1* is also the primary symbol for the unrelated gene anthocyanin 5-aromatic acyltransferase 1 (AT5G61160). Whilst it is outside our current scope, any symbols assigned to genes in *P. trichocarpa* could, of course, be applied to their orthologs in other plant species.

We are aware that, while our primary effort is focussed on naming protein-coding genes in the current *P. trichocarpa* reference assembly, there are, of course, non-coding RNA genes and additional genes that are present within the pangenome. Guidelines for naming genes in the human pangenome are in preparation and we intend to follow that strategy; this will be revisited when the *P. trichocarpa* pangenome is published.

## Conclusion

There is a clear need for more approved gene nomenclature in plants, as it provides a crucial ‘common language’ for anyone interested in genes. The utility of standardized naming has been shown many times in other kingdoms and species (McCarthy et al. 2023). We will implement a pragmatic approach to naming that uses published names where possible and involves the research community in decision-making. However, for a project like this to flourish, and especially to be extended to other species, it requires ongoing community support and funding. The process of gene naming, particularly where it involves expert data curation and negotiation with researchers, can be labour-intensive and time-consuming, though the costs involved are trivial compared with large-scale research projects. Unfortunately, funding for bioresources is extremely limited and the research community—including industry—will need to decide if the potentially highly significant time and funds saved by having standardized gene naming is something that they want to support in the long term.

## Data Availability

PGNC services are freely available at https://plant.genenames.org/. PGNC code is available at the GitHub repository (https://github.com/HGNC/pgnc-external-stack).
